# Zirconia in Dental Implantology: A Review of the Literature with Recent Updates

**DOI:** 10.3390/bioengineering12050543

**Published:** 2025-05-19

**Authors:** Sami Aldhuwayhi

**Affiliations:** Department of Restorative Dentistry and Prosthodontics, College of Dentistry, Majmaah University, Al Majmaah 11952, Saudi Arabia; s.aladdowihi@mu.edu.sa

**Keywords:** zirconia, dental implants, osteointegration, implantology

## Abstract

Zirconia dental implants have emerged as a transformative material in implantology, offering a biocompatible, esthetic, and durable alternative to traditional titanium implants. This comprehensive review explores the key properties of zirconia, including high fracture resistance, esthetic superiority, and low bacterial affinity. The ability of zirconia to integrate with bone through osseointegration, coupled with its resistance to plaque and inflammation, results in a product that is particularly suitable for patients with metal sensitivities or high esthetic demands. However, challenges such as brittleness and complex manufacturing processes persist. Advances in surface modification techniques and material optimization are poised to address these limitations, paving the way for broader applications. The purpose of this descriptive review was to emphasize the mechanical, antibacterial, osteointegration and survival rates of zirconia implants. This paper also summarizes findings from recent empirical studies, highlighting zirconia’s clinical performance, biological responses, and future potential as a mainstream implant material.

## 1. Introduction

Dental implantology has undergone significant advancements since its inception, with materials playing a pivotal role in its evolution. Titanium has long been regarded as the gold standard for dental implants due to its biocompatibility and mechanical properties. However, the need for alternatives has emerged due to concerns such as a metallic taste, potential hypersensitivity, and esthetic limitations in the anterior region. Zirconia, a ceramic material, has garnered attention as a promising alternative in dental implantology for its biocompatibility, esthetic superiority, and low bacterial affinity [1]. Zirconia implants, typically composed of yttria-stabilized tetragonal zirconia polycrystals (Y-TZP), offer several advantages. Their tooth-like color results in them being particularly appealing in cases where esthetics are paramount. Moreover, zirconia demonstrates high fracture resistance and wear properties comparable to titanium [2]. Unlike metallic implants, zirconia is non-conductive, reducing the risk of galvanic reactions and providing a biologically inert environment for surrounding tissues [3]. Around a 12% increase in the use of dental implants made up of zirconium has been reported [4]. Clinical studies have highlighted that zirconia implants can bond with bone-like apatite, allowing them to integrate with bone through osseointegration, comparable to titanium implants [5]. Additionally, zirconia’s low surface roughness and hydrophilicity contribute to reduced plaque accumulation and improved peri-implant health, addressing one of the significant concerns in long-term implant success [6]. However, its brittleness and manufacturing challenges necessitate further research and technological advancements to optimize its use. This review aims to comprehensively evaluate zirconia in dental implantology, focusing on its material properties, clinical performance, and associated biological responses. By summarizing the existing literature, it seeks to establish zirconia’s position as a viable alternative to traditional implant materials while identifying areas for future exploration in this rapidly advancing field.

## 2. Literature Review and Discussion

Zirconia dental implants have emerged as a promising alternative to titanium implants due to their superior biocompatibility, esthetic advantages, and resistance to corrosion and bacterial biofilm formation. Research consistently highlights zirconia’s ability to reduce inflammation and support better peri-implant health, making it an excellent choice for patients with sensitivity to metals or those prone to peri-implantitis [7]. Its natural tooth-like appearance also addresses the esthetic demands of modern dentistry, particularly in the anterior region [8]. Additionally, zirconia’s resistance to corrosion and lower thermal conductivity further solidify its potential as a durable and patient-friendly material [8]. Studies have shown significant advancements in zirconia implant designs, including surface modifications that improve osseointegration and mechanical performance [9,10]. Despite its benefits, zirconia presents challenges, such as brittleness under extreme loads and higher costs due to its complex manufacturing process [11]. Long-term evaluations of the survival rates and patient-reported outcomes indicate promising results, paving the way for zirconia to be a viable alternative in dental implantology [12]. Many studies have been conducted to examine the properties of zirconia for its use in dentistry.

**Biocompatibility:** Zirconia is highly biocompatible, meaning it interacts favorably with human tissues. It minimizes the risk of adverse reactions, inflammation, or rejection, making it suitable for individuals with metal sensitivities or allergies. Studies have shown its ability to promote soft tissue healing and maintain healthy peri-implant tissues. Studies have shown no significant differences in bone-to-implant contact and removal torque values compared to titanium implants; in fact, significantly better BIC values for acid-etched zirconia implants were reported compared to titanium implants [13]. Mostafa and Aboushelib [14] highlighted their exceptional bioactivity, chemical stability, and lower inflammatory response compared to titanium implants. They found that zirconia implants exhibited significantly higher bone-to-implant contact ratios and increased bone mass after implantation, indicating successful osseointegration. They also reported that the inflammatory response and bone resorption induced by zirconia were much lower compared to titanium particles. Furthermore, the enhanced biological responses observed with HA and PRP coatings on zirconia implants further support their biocompatibility.

**Esthetics:** Zirconia is naturally white, mimicking the color of natural teeth. This eliminates the risk of gum discoloration often associated with titanium implants, particularly in the cases of gingival recession. Its optical properties results in it being an ideal choice for anterior restorations. Borgonovo et al. [15] indicated that zirconia implants placed in areas with high esthetic value have shown encouraging survival rates (up to 100% after 13.5 months) and satisfactory esthetic evaluations, with average pink and white esthetic scores of 7.5 and 8, respectively. Alshehri, et al. [16] argued that zirconia hybrid abutment allowed for better optical effects that mimic natural teeth. In addition, zirconia implants provide a more natural appearance, reducing the risk of tissue discoloration often associated with metal abutments [17]. Spies et al. [18] presented patient-reported outcomes on zirconia implants, indicating high satisfaction levels, particularly regarding esthetics. Participants reported significant improvements in function and esthetics after treatment, reflecting a positive perception of zirconia implants. Overall, the findings suggested that zirconia implants effectively meet patient needs, especially in esthetic considerations.

**Mechanical Properties:** Zirconia’s mechanical strength has been widely studied, and it stands out as a durable material for dental implants due to its high fracture toughness, flexural strength, and fatigue resistance. Palmero et al. [19] developed an innovative zirconia-based composite with Al_2_O_3_ and SrAl_12_O19 in a ceria-stabilized zirconia matrix. They employed a novel surface coating method for the precise control of microstructural features and achieved fully dense materials with optimal phase distribution. The ceria content significantly affected the aging kinetics; 10.5 mol% ceria showed stability against low-temperature degradation. Hence, they showed that zirconia-based composites exhibited excellent mechanical properties, including high strength and toughness, making them suitable for dental applications. The innovative synthesis method allowed for the precise control of microstructural and compositional features, resulting in fully dense and stable materials. Additionally, the presence of ceria in the composites improved their resistance to aging, enhancing their long-term stability in biomedical applications. Liao et al. [4] examined the corrosion behavior of different Zr–Sn–Nb zirconium alloys compared to the Zr-4 alloy in superheated steam at temperatures between 390 and 455 °C and a pressure of 10.3 MPa. They identified new corrosion and transition phenomena and created a new model for predicting the transition kinetics over a broad temperature range. The time taken for all the alloys to change showed a relationship similar to the Arrhenius equation with the temperatures they were exposed to. The weight gain during the transition and the thickness of the oxide layer increased as the temperature increased. A significant increase in the transition oxide thickness was observed for the Zr-4 alloy between 390 and 455 °C. Gutiérrez Robledo et al. [20] tested a new block design that incorporated a zirconia insert bonded to a resin composite, which aimed to mask the grayish color of the titanium abutments and ensure optimal fit tolerance. This study evaluated the mechanical resistance and fracture behavior of zirconia in comparison to other materials, highlighting its role in providing strength and durability under static and dynamic loads. Thomas et al. [10] found that the combination of zirconia with resilient materials could help absorb mechanical loads, potentially reducing stress on the crown–abutment connection and preventing catastrophic failures. The authors also observed that while zirconia regions exhibited standard hardness and fracture-toughness values, the composite regions showed lower hardness, indicating a difference in material properties that could influence their application in dental restorations. The mechanical properties of zirconia are affected by prolonged exposure to an aqueous medium, with failure often linked to fractures. Future studies should focus on mechanical loading in a simulated oral environment to understand the interplay between mechanical and electromechanical properties. A systematic review by Attard et al. [21] found that zirconia implants, both one-piece and two-piece, can withstand masticatory forces for several years, with in vitro studies indicating they can endure a loading duration of 10 million cycles at 95 N force. However, the bending moment to fracture (BMF) of the zirconia implants was significantly less than that of the titanium implants, raising concerns about their fracture resistance. Additionally, this study highlighted a lack of long-term clinical evidence specifically addressing the mechanical strength of zirconia implants.

**Surface Characteristics:** Surface roughness is a critical factor in achieving successful osseointegration. Micro-roughened and nano-structured zirconia surfaces improve the adhesion, proliferation, and differentiation of osteoblasts.

**Figure 1:**
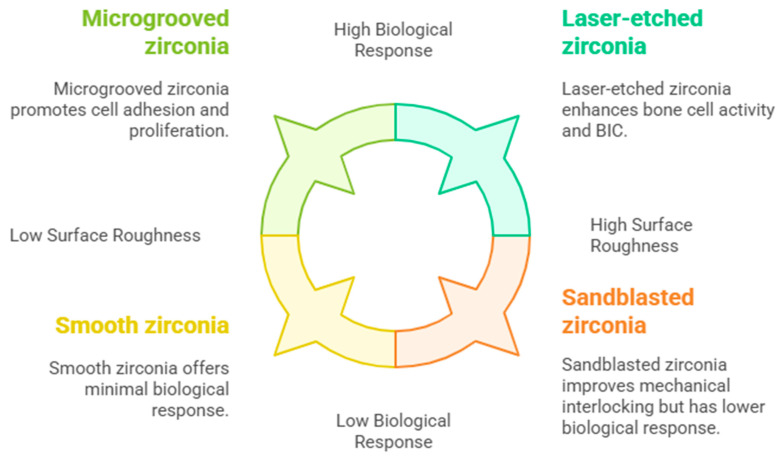
Surface characteristics of zirconia implants.

Jin et al. [9] demonstrated that surface modifications, such as sandblasting and laser etching, enhanced the biological response of zirconia implants by promoting bone cell activity and improving mechanical interlocking between the implant and bone tissue. Micro-textured zirconia implants have been shown to enhance bone-to-implant contact (BIC), leading to improved stability and accelerated healing compared to smoother surfaces. The surface modifications, particularly those that increase roughness, play a crucial role in promoting osseointegration, which is vital for the success of dental implants. Studies indicated that rough surfaces, such as those modified by laser etching, exhibit higher BIC and improved biological responses, including increased osteopontin expression, which is essential for bone formation [9]. Micro-/nano-structured porous zirconia surfaces have demonstrated superior cell affinity and osteogenic differentiation, significantly enhancing bone matrix development in vivo [22]. Microgroove patterns on zirconia surfaces have been shown to promote cell adhesion and proliferation, reducing stress on surrounding bone tissue, which contributes to better osseointegration [23]. A prospective study highlighted that the surface topography of zirconia implants affects the first BIC, with moderately rough surfaces yielding favorable outcomes [11]. These surface characteristics are illustrated in Figure 1 based on the published literature.

**Figure 2:**
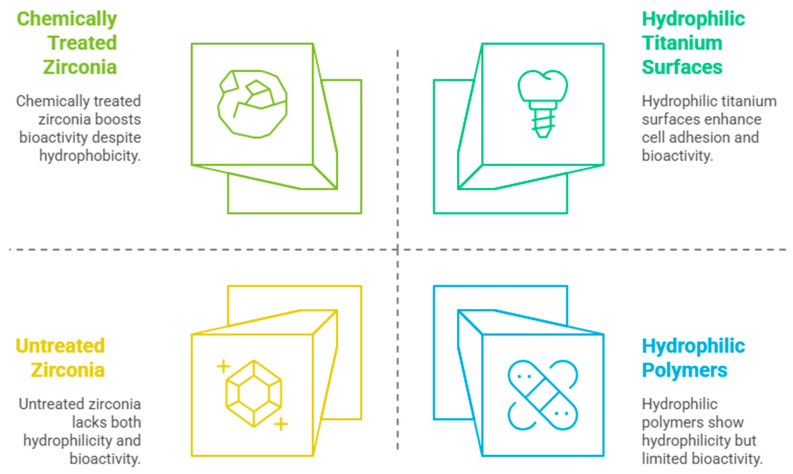
Hydrophilicity and wettability of zirconia implants.

**Hydrophilicity and Wettability:** Hydrophilic surfaces facilitate protein adsorption and cell attachment, accelerating the early stages of osseointegration [24]. Studies indicate that hydrophilic surfaces promote fibroblast and osteoblast adhesion, leading to improved cell viability and differentiation [25]. The combination of hydrophilicity and nanostructuring on titanium surfaces has been linked to shortened healing times [25]. Tuna et al. [26] found that chemically treated zirconia, such as hydrogen-peroxide-modified surfaces, significantly increased wettability and osteogenic activity, improving early implant stability. Rohr et al. [27] confirmed that hydrophilic zirconia surfaces showed enhanced bioactivity and reduced soft tissue encapsulation, contributing to better integration. Noro et al. [28] found that the more hydrophilic surfaces of zirconia led to greater cell adhesion during the initial stages of osseointegration. Cells spread more effectively on hydrophilic surfaces compared to hydrophobic ones, which indicated an improved biological response. The hydrophilicity and wettability of the zirconia implants are illustrated in Figure 2. Furthermore, surface modifications resulted in superhydrophilicity, which was maintained even after immersion in aqueous solutions, crucial for clinical applications in implants.

**Antibacterial Properties:** Zirconia exhibits notable antibacterial properties, making it an excellent material for dental implants. These properties are largely attributed to its smooth surface, low surface energy, and the ability to resist biofilm formation. Unlike titanium, which may facilitate bacterial adhesion under certain conditions, zirconia’s unique characteristics reduce the risk of peri-implantitis and other infections [29]. Zirconia surfaces are less conducive to bacterial attachment compared to roughened or metallic surfaces like titanium. This is primarily due to its smooth and chemically inert surface. The findings of Chopra et al. [30] indicated that nano-engineered zirconia (ZONs) demonstrated the enhanced bioactivity of osteoblasts while not promoting the formation of salivary polymicrobial biofilms. Overall, the modified zirconia surfaces were proposed to improve tissue integration without increasing biofilm formation. It has been observed that zirconia implants accumulated significantly lower levels of biofilms than titanium implants in early stages [31]. The review paper by Sivaraman et al. [32] presented several findings regarding the biofilm accumulation on zirconia implants compared to titanium implants. Zirconia implants were observed to accumulate significantly lower levels of biofilms than titanium implants which was attributed to their bio-inert properties, which enhance periodontal integration and reduce inflammation around the implants. Zirconia implants exhibited a reduced number of cocci and rods, with a notable increase in levels of Streptococcus mutans and a decrease in Streptococcus sanguis compared to titanium implants. More specifically, in a randomized clinical trial, it was found that the presence of Streptococcus mutans was recorded in 58.34% of samples from zirconia implants, while Porphyromonas gingivalis was detected in 100% of samples from cast and polished titanium, highlighting the lower pathogenic presence on the zirconia implants.

**Figure 3:**
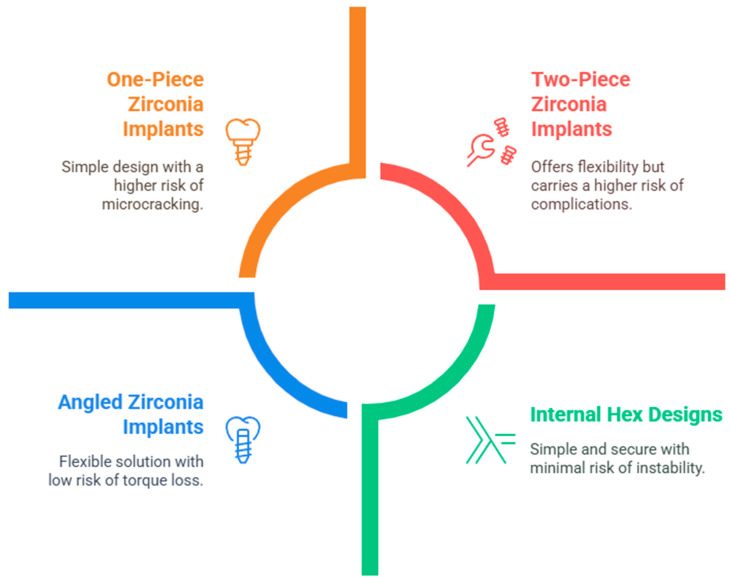
Types of zirconia implants.

**Types of Zirconia Implants:** There are different types of zirconia dental implants, each designed to meet specific patient needs and clinical preferences. These implants vary in terms of design, material composition, and intended application. Below are the primary types of zirconia implants currently available, and the types of Zirconia implants are illustrated in Figure 3.

**One-Piece Zirconia Implants:** These implants have a single-piece design, meaning the implant and abutment are integrated into one continuous structure. This design is often preferred for its simplicity and enhanced strength, reducing the risk of complications related to screw loosening [33]. CeraRoot offers one-piece zirconia implants that are designed for esthetic and functional advantages, especially for patients with metal allergies or sensitivities [34]. Z-Systems also provides one-piece zirconia implants, which are known for their durability and resistance to plaque accumulation [35]. However, it has also been highlighted that Z-Systems implants are particularly susceptible to microcracking due to their manufacturing process, which includes laser treatment. This susceptibility may affect their long-term performance despite their initial advantages.

**Two-Piece Zirconia Implants:** Two-piece systems feature a separate implant body and an abutment. These implants offer more flexibility in terms of adjusting the angle and position of the abutment after implantation. A study from Cionca et al. [36] presented a prospective clinical study evaluating this two-piece zirconia implant system, specifically designed to support all-ceramic crowns in partially edentulous patients. A total of 49 two-piece zirconia implants were placed in 32 systemically healthy patients, with a follow-up period ranging from 369 to 889 days after loading. The cumulative survival rate for the implants was reported to be 87% one year after loading, with all failures attributed to aseptic loosening, and no implants lost after the first year. The study also noted a cumulative soft tissue complication rate of 0%, indicating excellent biocompatibility and stability of the soft tissues around the implants. Additionally, this study highlighted that the two-piece system allowed for the easy unscrewing of implants without causing bleeding or suppuration, ruling out peri-implantitis as a cause of failure. Astra Tech Implant System provides zirconia alternatives to its popular titanium implants. Altarawneh et al. [37] discussed the use of the Astra Tech Implant System, specifically mentioning various implant sizes such as AstraTech 4.0 S × 8 mm and AstraTech 4.5 × 9 mm, utilized in the treatment of edentulism. It highlights the placement of the cover screws on maxillary implants and the healing abutments on mandibular implants during the surgical phases. The system is noted for its compatibility with advanced technologies like CAD/CAM for creating fixed prostheses, contributing to successful clinical outcomes.

**Internal and External Hex Designs:** In the internal helix, a hexagonal connection is present inside the implant, offering a stable and secure fit with the abutment. It is the most common connection used in modern dental implants. This design provides a stable and secure fit between the implant and the abutment, enhancing the overall stability of the dental restoration. The secure connection may contribute to improved longevity and performance of the dental implant system. In the external implant, the hexagonal connection is present on the outside of the implant, which provides a robust connection for the abutment but can be less stable compared to the internal hex design. Both types are available in zirconia implants, although internal hex designs are typically more popular for their precision and durability. Zirconia’s tooth-like color provides a more natural appearance, making it a preferred choice for visible areas in dental restorations.

**Angled Zirconia Implants:** Angled zirconia implants have emerged as a viable solution for addressing anatomical challenges in dental implantology, particularly in esthetic zones. Research indicates that these implants can maintain adequate torque and fracture resistance, making them suitable for various clinical scenarios. The following sections detail their performance, advantages, and considerations. Studies show that monolithic zirconia restorations with angulated screw channels exhibit satisfactory fracture strength, particularly at 15° angulation, which outperformed 25° angulated counterparts [38]. Torque loss was found to be insignificant across different angulations, suggesting stability in clinical applications [38]. A retrospective study indicated minimal marginal bone loss around angled implants, with an average of 0.66 mm on buccal and 0.93 mm on lingual surfaces, demonstrating their reliability over 15 to 30 months [39]. This study reported only one implant failure among fifty-eight, highlighting the overall success of angled implants in clinical settings [39]. The integration of digital workflows in the design and fabrication of restorations for angled implants enhanced precision and adaptability, allowing for improved outcomes in complex cases [40]. For example, Z-Systems offers angled zirconia implants to cater to specific anatomical needs.

**Osseointegration and soft tissue integration of zirconia implants:** Osseointegration is a dynamic healing process in which an implant is biologically fixed to the living bone without any connective tissue in between [41,42,43,44]. Zirconia is a biomaterial that is beneficial for soft tissue health and osseointegration due to its non-toxic, non-corrosive properties and its ability to conduct electricity in bone [45]. Research has shown that zirconium is biocompatible, hence it does not promote cancer in muscle and bone implants [46,47,48,49,50,51]. The implant thread, which touches the bone, offers the main stability following implantation. Water molecules form a pellicle that enables intracellular matrix proteins to adhere to the surface of the implant. Cells find it simpler to adhere together, migrate, and differentiate because of this set up [52]. A blood clot between the osteotomy site and the implant triggers the release of inflammatory cells and other growth factors. This procedure promotes stem cells to migrate to the implant surface and new blood arteries to form [53,54,55]. Although titanium could be biocompatible, some people nonetheless run the risk of problems like infection or rejection. Additionally, the presence of a blood clot between the implant and osteotomy site may increase the risk of inflammation and delay the healing process. Surface characteristics of the implant biomaterial, including the composition, surface topography, and surface roughness, may influence how the wound heals following the implant placement and play a critical role in osseointegration [56,57]. Osteoblast cells were placed on two distinct kinds of roughened zirconia substrates in a laboratory experiment. In less time, both produced a positive cellular response, or cell adhesion. The average BIC for animal model research employing loaded titanium and loaded zirconium implants was 72.9% for the titanium implants and 67.4% for the zirconium implants [56,57,58]. Although the soft tissue around the implants was of the same size, this result implied that both kinds of implants exhibited the same degree of osseointegration. Clinical trials have also shown that zirconia implants were successful in maintaining their location and raising their survival rate [59,60,61]. Overall, both zirconia and the gold standard material, titanium, osseointegrate to a similar degree; zirconia has been proven to be superior in notable situations [62]. These results suggest that in dental implant operations, zirconia implants may be reasonable substitutes for titanium implants. Results from both animal and clinical research indicate that zirconia implants have osseointegration rates equivalent to those of titanium implants, with some instances even indicating zirconia to be better. These data back up the usage of zirconia implants as a consistent choice for patients wanting dental implant therapy. Further study and long-term investigations are required to properly evaluate the efficacy and lifetime of zirconia implants compared to titanium implants. The proper integration of tissue, which helps to preserve the bone underneath and keeps bacteria away, depends on osseointegration and the seal of the gum tissue surrounding the dental implants. Factors include implant surface roughness, surface roughness techniques, and abutment material that affect soft tissue response [63,64,65,66]. Research indicates that titanium implants cause higher peri-implant mucosal loss than zirconia implants with altered surfaces [67,68,69]. Zirconia implants are bio-inert, hence preventing bacterial cell attachment and biofilm development. In vitro experiments have shown that while zirconia surfaces stop bacterial attachment, they let cells attach and proliferate [70].

The presence of zirconium is intuitive, as it is a constituent material of the implant itself. This evidence suggests that the implant can withstand stresses without catastrophic failure. Because bone apposition occurs on the implant surface, the specimen contains calcium, phosphorus, and oxygen. These are the main elements that comprise the bone mineral matrix. However, the mechanical properties of zirconia implants depend on factors such as manufacturing process, surface treatment, and volumetric expansion. In the past, companies made zirconia implants by cutting away material, which caused changes in the structure and created stress. To avoid these phase changes during manufacturing, ceramic injection molding and sintering are used. The micro-roughness profile of the zirconia implant shows holes 10–20 μm in size and material deposition, which aligns with Thomé et al.’s findings and suggests good potential for cellular adhesion and osseointegration. The survival rate of zirconia two-piece implants ranges between 83% and 99%. However, a study found a lower bone–implant contact rate compared to the titanium implants, contradicting the rest of the literature. A higher failure rate for zirconia two-piece implants outside the reported range could challenge the conclusion that it has good potential for osseointegration. Factors such as patient responsibility, treatment planning, implant surface, and design could contribute to clinical failure. Long-term studies are needed to assess the clinical performance of two-piece zirconia implants and investigate the potential benefits of surface modifications and different loading protocols on success rates. Understanding the reasons for implant failure is crucial for improving the success rates of zirconia implants. By conducting thorough research and long-term studies, clinicians can gain valuable insights on how to enhance the clinical performance of these implants. Exploring surface modifications and adjusting loading protocols may offer new possibilities for increasing the success rates of zirconia implants and improving patient outcomes in the future.

**Figure 4:**
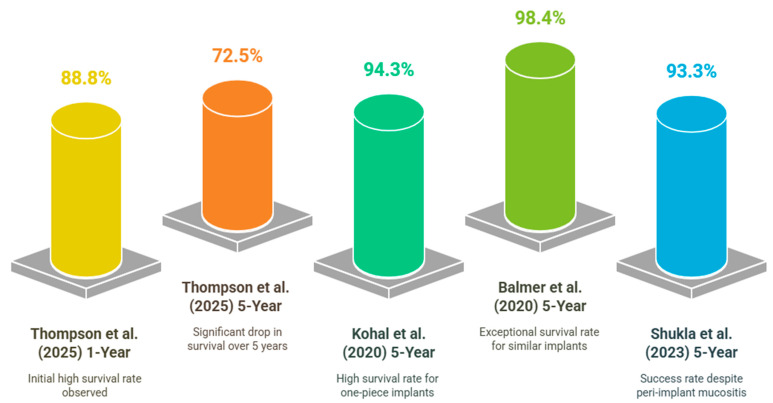
The survival rates of zirconia implants based on published literature [71,72,74,76].

**The Survival rate of Zirconia implants:** The long-term survival rates of zirconia implants, particularly when comparing 1-year [71,72] and 5-year data [73,74,75,76,77,78,79,80], reveal a nuanced picture of their durability and clinical performance (Figure 4). While zirconia implants show promising survival rates, especially in the short term, the decline over five years highlights the need for careful patient selection and the management of modifiable risk factors. The variability in survival rates across studies suggests that further research is needed to optimize implant design and address technical complications. Moreover, the high esthetic and biocompatible advantages of zirconia implants continue to support them as a favorable choice, despite the challenges in achieving long-term success [73,74,75,76]. Zirconia implants are increasingly favored for their esthetic and biocompatible properties, yet their long-term success rates vary across different studies. Although the survival rates are typically high at one year, there is a noticeable decline over five years due to elements like implant design, patient-specific variables, and technical difficulties. A detailed analysis of the survival rates is based on the provided studies. Zirconia implants demonstrate a high survival rate in the first year based on a systematic review. For instance, a systematic review reported a survival rate of 87.7% for one-piece zirconia implants after at least a year of function [71]. In a study focusing on zirconia-implant-supported fixed complete dentures, the 1-year survival rate was 88.8% [72]. Over five years, the survival rates of zirconia implants tend to decrease. For example, the same study that reported an 88.8% survival rate at one year showed a decline to 72.5% at five years [72]. A few studies reported higher 5-year survival rates. A prospective cohort study found a 94.3% survival rate for one-piece zirconia implants [73], while another study reported a 98.4% survival rate for similar implants [74]. Common reasons for failure included framework fractures and implant loss. Another study noted that the most frequent complication was peri-implant mucositis, occurring in 16.7% of implants, with a 5-year success rate of 93.3% [75]. These studies suggested that while the overall survival rate for dental implants remained high, there was variability depending on the type of implant used.

One-piece zirconia implants appear to have higher success rates compared to other types of implants. However, it is important to consider the common reasons for failure, such as framework fractures and implant loss, when evaluating long-term success. Additionally, addressing and managing peri-implant mucositis is crucial for maintaining the overall success of dental implants. Regular maintenance and follow-up appointments are essential in preventing peri-implant mucositis and catching any potential issues early on. Proper oral hygiene practices, including daily brushing and flossing, can also contribute to the longevity of dental implants. We should educate patients on the significance of these factors to guarantee the long-term success of their implants. By addressing these key points and staying proactive in their oral health care, individuals can maximize the benefits of their dental implants and enjoy a healthy, functional smile for years to come. The design of the implant and the type of zirconia used can significantly impact survival rates. Monolithic zirconia restorations, for instance, demonstrated a perfect survival rate of 100% over five years, compared to 95.8% for layered restorations [76]. Factors such as smoking and the condition of the opposing dentition have been associated with increased failure rates [77]. Additionally, technical complications like chipping and occlusal roughness are prevalent, affecting long-term outcomes [78,79,80]. While the type of zirconia used may impact survival rates, other factors such as patient habits and technical complications also play a significant role in the longevity of dental implants. It is important to consider a holistic approach to ensure the success of the restoration in the long term.

**Further aspects:** Zirconia dental implants are expected to become a mainstream choice in dental implantology due to their unique properties, including biocompatibility, esthetics, mechanical strength, and resistance to plaque. Zirconia implants are biocompatible, have a low risk of peri-implantitis, and are suitable for long-term applications, especially in patients with metal sensitivities or allergies. Surface treatments like sandblasting, acid etching, and laser texturing are expected to result in the zirconia being more hydrophilic and better at integrating with bone. Future research may focus on creating tailored surfaces to improve implant stability and bone integration even in patients with compromised bone quality. Zirconia implants are highly esthetic due to their tooth-like color and ability to prevent dark gingival shadows associated with titanium implants. Patients can easily distinguish between zirconia implants and natural teeth, resulting in high pink and white esthetic scores (PES and WES). Zirconia’s resistance to plaque accumulation is expected to reduce the incidence of peri-implant diseases. Bioactive coatings with antimicrobial agents could be added to future designs to further enhance this property. As 3D printing and CAD/CAM technologies improve, customization will enhance patient outcomes and satisfaction. Zirconia implants are increasingly being evaluated for full-mouth reconstructions due to their mechanical strength and esthetic appeal. A digital workflow allows for the accurate and efficient fabrication of restorations, resulting in high patient satisfaction and minimal complications. Despite some problems, new developments in material composition, surface modifications, and manufacturing methods are quickly addressing these issues. Zirconia implants seem to have similar or even better clinical outcomes than titanium implants in terms of survival rates, patient satisfaction, and health around the implant.

## 3. Conclusions

Zirconia implants represent a significant advancement in dental implantology, addressing key challenges associated with titanium while meeting the growing demand for metal-free, biocompatible solutions. Their natural tooth-like appearance, resistance to corrosion and biofilm formation, and excellent osseointegration capabilities underscore their suitability for a wide range of clinical applications. As advancements in 3D printing, bioactive coatings, and digital workflows continue, zirconia implants are poised to redefine the future of restorative dentistry, offering tailored, durable, and esthetically superior solutions for patients worldwide.

## Data Availability

No new data were created or analyzed in this study.
